# The relationship between Google search interest for pulmonary symptoms and COVID-19 cases using dynamic conditional correlation analysis

**DOI:** 10.1038/s41598-021-93836-y

**Published:** 2021-07-13

**Authors:** Halit Cinarka, Mehmet Atilla Uysal, Atilla Cifter, Elif Yelda Niksarlioglu, Aslı Çarkoğlu

**Affiliations:** 1 Yedikule Training and Research Hospital for Chest Diseases and Thoracic Surgery, University of Health Sciences Turkey, Istanbul, Turkey; 2 Department of Economics, Altinbas University, Istanbul, Turkey; 3 Department of Psychology, Kadir Has University, Istanbul, Turkey

**Keywords:** Fever, Epidemiology, Respiratory signs and symptoms

## Abstract

This study aims to evaluate the monitoring and predictive value of web-based symptoms (fever, cough, dyspnea) searches for COVID-19 spread. Daily search interests from Turkey, Italy, Spain, France, and the United Kingdom were obtained from Google Trends (GT) between January 1, 2020, and August 31, 2020. In addition to conventional correlational models, we studied the time-varying correlation between GT search and new case reports; we used dynamic conditional correlation (DCC) and sliding windows correlation models. We found time-varying correlations between pulmonary symptoms on GT and new cases to be significant. The DCC model proved more powerful than the sliding windows correlation model. This model also provided better at time-varying correlations (r ≥ 0.90) during the first wave of the pandemic. We used a root means square error (RMSE) approach to attain symptom-specific shift days and showed that pulmonary symptom searches on GT should be shifted separately. Web-based search interest for pulmonary symptoms of COVID-19 is a reliable predictor of later reported cases for the first wave of the COVID-19 pandemic. Illness-specific symptom search interest on GT can be used to alert the healthcare system to prepare and allocate resources needed ahead of time.

The new Coronavirus (SARS-CoV-2) causing Coronavirus disease 2019 (COVID-19) was first detected in Wuhan, China, in December 2019 and rapidly spread worldwide^1^, reaching a pandemic status by March 11, 2020^2^. Europe was the second epicenter of the pandemic after Wuhan and continues to struggle with controlling the spread and fatalities due to COVID-19. As of December 2020, the World Health Organization (WHO) reported more than 70 million confirmed cases and close to 1.6 million deaths globally; Europe reported close to 22 million confirmed cases and an excess of 450 thousand fatalities^3^.

National responses and the ability to monitor and control the pandemic varied significantly, especially during the first months^4^. With the implementation of social distancing regulations, millions turned to the internet to find answers to their questions and worries about the pandemic; between March and May 2020, Coronavirus related searches became the most popular search terms on Google^5^.

Google Trends (GT) is a web-based, innovative tool made available by Google to analyze the content, frequency, and popularity of search queries in Google search across various regions and languages. Analysis can be carried out within a given timeframe, focused on a particular public event such as the onset of an epidemic^6^.

There is a growing body of research demonstrating the use of GT that monitoring online queries via GT correlates with specific behavioral outcomes. Previous studies using various methods analyzed the relationship between GT search interest and suicide rates, infectious diseases transmissibility, and the spread trajectory of emerging new pathogens such as SARS, Ebola, and 2009 influenza^7–10^. Various studies focusing on the public search interest on the web revealed GT search interest monitoring for symptom searches to be valuable in identifying new cases in COVID-19 pandemics^11–13^.

There is also some disagreement in the field about the validity of using Google Trends as a tool for digital epidemiology^14–16^. GT data can be influenced by many factors: historical events, public interest, or media coverage. However, when we study the relationship of this fragile data with actual data such as daily confirmed COVID-19 cases, the resulting correlation can be more reliable. Thus, monitoring this relationship can be a viable tool for understanding the movement of the pandemic.

Lippi et al. investigated the capacity of Google search volume of symptoms such as fever, cough, and dyspnea to predict the trajectory of the early 2020 COVID-19 outbreak in Italy using Spearman's correlation method. They concluded that GT's continuous monitoring is a valuable instrument in the early detection of COVID-19 outbreaks^12^. Most studies used conventional correlation methods to determine the relationship between symptom search and cases^12,17–19^. Other studies employed moving average (MA) methods to smooth daily fluctuations of symptoms and later new case emergence, and they selected three to seven days as their moving average^20,21^.

Some authors also preferred shifting the symptom search results to match the GT search and new cases^21–23^. One common denominator in all these studies was the use of non-dynamic statistical procedures. Another approach is to use wave analysis to detect the co-movement between symptoms and cases^24^. However, this approach has the limitation of not seeing correlation over time.

Asseo et al. relied on sliding windows correlations, a straightforward time-varying approach to assess the relationship between taste and smell loss on GT, and emerging case numbers. The sliding windows correlation method allows for monitoring correlations for each time period separately but still uses Pearson correlations^25^. Asseo et al.'s approach carries the limitation of conventional correlation, which lacks the ability to work with time-varying co-movement. On the other hand, the DCC model considers both time-varying correlation and time-varying variances, and this method is more powerful than the conventional correlation methods, including sliding windows with Pearson or Spearman correlation analysis^26^.

The DCC model, developed initially for financial time series, has been used by several researchers in finance and neuroscience. In finance, several studies used the method to investigate Google search interest and financial market behaviors^27–29^. In neuroscience, Lindquist et al. used the DCC model to study the time-varying correlation among several brain signals in functional magnetic resonance imaging (fMRI). The authors concluded that the DCC model better captured time-varying correlations as it minimizes random noise in the estimations^26^. We believe the DCC model can also be used in health sciences to capture the time-varying relationship between symptom search and new case emergence.

We aim to present DCC as a model that better fits the time-lagged nature of our data set and compare its viability against the sliding window correlation method to study the relationship between searches of fever, cough, and dyspnea on GT and new cases in Turkey, Italy, Spain, France, and the UK.

## Methods

### Data

Google search interest trends are calculated by dividing the number of queries of interest by the total number of queries for all search terms over the same time and region. Each query share is normalized on a scale of 0 to 100, with 100 representing the share's maximum value for the period and region selected. The scaled query share values are plotted daily, generating a time series. Search terms included pulmonary symptoms, e.g., fever, cough, dyspnea, as previously reported to be associated with COVID-19 infection [15 Lippi]. Searches for these terms covered Turkey, Italy, Spain, France, and the United Kingdom (UK), the European countries most affected by the COVID-19 pandemic. We have focused on these countries due to differences in geographic locations, cultures, and health systems.

Furthermore, the first wave of the pandemic presented at different times across these countries. At the same time, similar precautionary measures such as the shutdown of all schools and universities, closure of museums, cultural centers, cinemas, theatres, pubs, and the suspension of international flights were undertaken in all countries at approximately the same time^30,31^.

Google searches for pulmonary symptoms were obtained by R X64 40.2(R: A Language and Environment for Statistical Computing) using "gtrendsR" package for the dates between January 01 and August 31, 2020. Search terms were determined in Turkish and were later translated to the relevant languages (Italian, French, Spanish, and English) via Google Translate, and then checked for accuracy by native speakers. We used "fever," "cough" and "dyspnea" or "shortness of breath" as search terms for pulmonary symptoms ("ateş", öksürük", "nefes darlığı" for Turkish, "febbre ", tosse" and "dyspnée" for Italian, "fièvre", "toux” and "essoufflemen" for French, "fiebre", "tosse" and "dyspnea", for Spanish). Each term was searched, selecting “all categories” for each particular country. The search was conducted on September 1, 2020. We obtained the data of new cases for each country from the WHO COVID-19 database^3^.

### Statistical analysis

**Figure 1:**
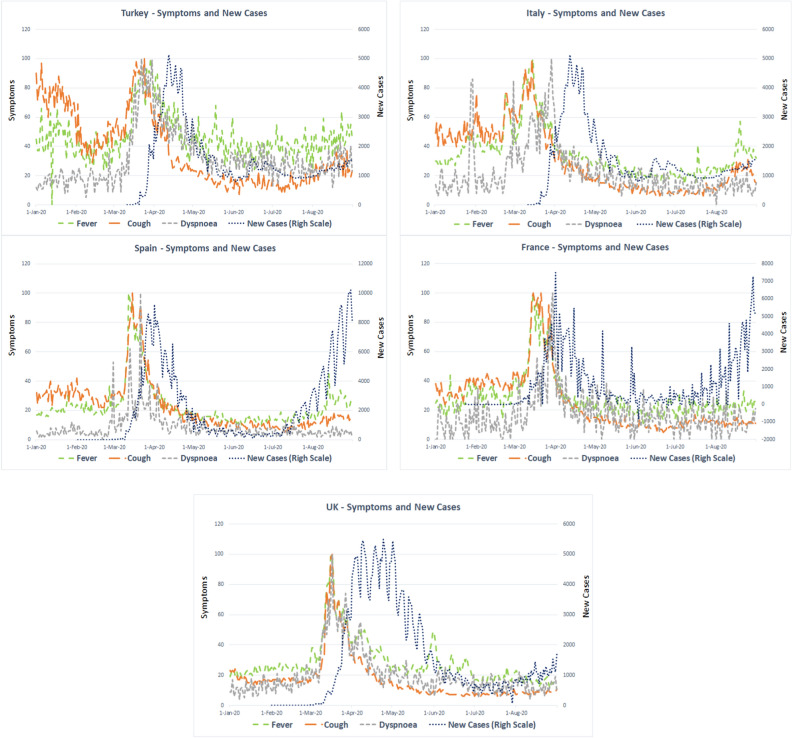
Symptoms and cases in raw data.

An initial check of the raw data revealed very high fluctuations and time lags between symptoms and new cases (see Fig. 1). Previous studies used a 3- to 7-day moving averages^20,21,32^ to transform the data. We analyzed our data using various moving averages ranging from 3 to 7 days to deal with the high fluctuations and observed that five days was most appropriate to smooth the data. Next, we shifted symptom search results forward to capture the time lag between symptom searches and new case reports. We realized that each symptom in each country needed a unique time period. We used the RMSE approach to determine the best fit period for each symptom in each country. Symptoms were shifted forward until the minimum RMSE was observed. We used the sliding windows correlation offered by Asseo et al. The authors selected a time frame of 31 days and rolled the correlation with one day^25^. We deployed the same method and calculated sliding window correlations for the raw data, moving average, and shifted data.

We later carried out the DCC model to understand the dynamic correlation between Google search interest for the three identified pulmonary symptoms and new cases. The DCC method, originally proposed by Engle et al.^33^ has been adapted to different multivariate cases by Tse et al.^34^. This method was developed for conditional volatility in financial portfolios, and it has been used in interdisciplinary fields^26^. The DCC model proved more powerful than the Pearson, Spearman's rank, or constant conditional correlation model as it captures dynamic correlations between two-time series (For a detailed description of the method, refer to Tse et al.). We analyzed the data using Oxmetrics 8 software and Microsoft Excel Microsoft, Redmond, WA, United States), and a p-value < 0.05 was considered to indicate as statistically significant difference.

## Results

**Figure 2:**
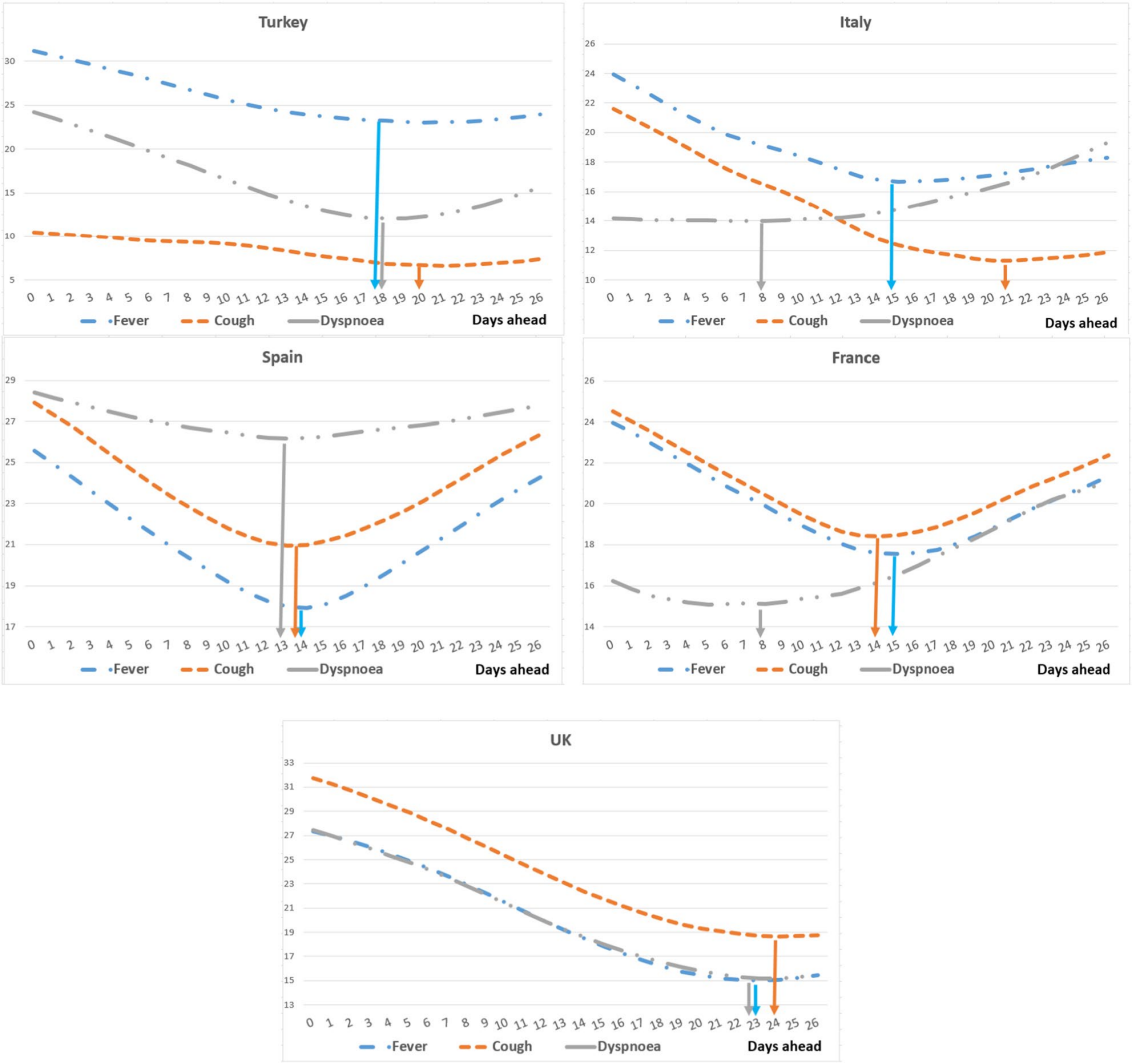
Root mean squared errors of symptoms' shifts.

We initially checked the normality of the data using the Shapiro–Wilk test and observed that not all the series were normally distributed. Therefore, we used Spearman correlations instead of Pearson correlations for the rest of our analyses. The Spearman correlations for raw data and five days moving averaged and shifted data are presented in Table 1. When we examined the raw data, we found correlation coefficients to be weak (less than 50%) and/or non-significant for most symptom searches and cases*.* We first transformed the observations to a five-day moving average, then shifted symptoms separately with the RMSE values. We found that Spearman correlation coefficients between symptoms and new cases increased to moderate levels and became significant at p < 0.01 (see Table 1 for symptom-specific p-values). Figure 2 shows the RMSE values for fever, cough, dyspnea in five countries. The arrows show the optimum shift days where the RMSE values are minimum. Table 2 lists the symptom search shifts for each symptom in each country. We observed that the optimum time lag for each symptom ranged from 8 to 24 days. These findings show that search terms on GT may need to be shifted separately to better fit the nature of the phenomenon at hand.

**Table 1:** Spearman correlations between symptoms and new cases.

	Turkey	Italy	Spain	France	UK
**Fever**					
Raw data	0.243 (0.001)	0.295 (0.000)	0.378 (0.000)	0.017 (0.804)	0.257 (0.000)
MA and shifted data	0.498 (0.000)	0.899 (0.000)	0.653 (0.000)	0.633 (0.000)	0.630 (0.000)
**Cough**					
Raw data	0.184 (0.015)	0.252 (0.000)	0.082 (0.235)	-0.004 (0.955)	0.096 (0.162)
MA and shifted data	0.594 (0.000)	0.865 (0.000)	0.339 (0.000)	0.655 (0.000)	0.671 (0.000)
**Dyspnea**					
Raw data	0.359 (0.000)	0.479 (0.000)	0.238 (0.000)	0.242 (0.000)	0.291 (0.000)
MA and shifted data	0.732 (0.000)	0.781 (0.000)	0.307 (0.000)	0.382 (0.000)	0.744 (0.000)

*Notes:* Symptoms and cases are estimated with five days moving average, and symptoms are shifted with days with minimum RMSE values. p-values are shown in parentheses.

**Table 2:** Symptom search shifts with minimum root mean squared errors (RMSE) (days).

	Turkey	Italy	Spain	France	UK
Fever symptom	18	15	14	15	23
Cough	20	21	14	14	24
Dyspnea	18	8	13	8	23

Before estimating the time-varying correlation, we checked the dynamic conditional correlation versus constant correlation using two diagnostics: the E-S and the LM tests. These tests use Chi-square fit values and check if dynamic conditional correlation should be used rather than constant correlation. Table 3 shows that the constant correlation hypothesis should be rejected for all of the series at p < 0.01. We suggest using a time-varying correlation to monitor the co-movement of symptom search and new cases emergence.

**Table 3:** Diagnostic checks of the DCC model.

	Fever symptom—New cases	Cough symptom—New cases	Dyspnea symptom—New cases
**E-S tests**			
Turkey	60.812 (0.000)	190.896 (0.000)	63.502 (0.000)
Italy	56.487 (0.000)	61.121 (0.000)	62.935 (0.000)
France	44.434 (0.000)	86.255 (0.000)	64.142 (0.000)
Spain	126.420 (0.000)	163.946 (0.000)	80.009 (0.000)
UK	99.660 (0.000)	133.527 (0.000)	90.217 (0.000)
**The LM Test**			
Turkey	32.666 (0.000)	32.121 (0.000)	32.241 (0.000)
Italy	35.621 (0.000)	45.185 (0.000)	37.981 (0.000)
France	27.076 (0.000)	28.461 (0.000)	27.076 (0.000)
Spain	27.275 (0.000)	27.595 (0.000)	27.597 (0.000)
UK	39.914 (0.000)	38.426 (0.000)	43.170 (0.000)

E-S Test_(j)_ ~ χ^2^_(j+1)_ and LM test ~ _(j)_ ~ χ^2^_(N*+(N-1)/2))_ under H_0_: Constant correlation, H_1_: Dynamic correlation. p-values are shown in parentheses.

Table 4 reports the DCC coefficients for the relationship between pulmonary symptom searches and new case emergence in Turkey, Italy, Spain, France, and the UK. We found that significant, moderate to high DCC correlations. The correlation degree of pulmonary symptom search was different for each of the symptoms and countries. The findings demonstrate that the null hypothesis of constant correlation should be rejected. (p < 0.01).

**Table 4:** DCC between symptoms and cases.

	Fever symptom—New cases	Cough symptom—New cases	Dyspnea symptom—New cases
Turkey	0.822 (0.000)	0.869 (0.000)	0.663 (0.004)
Italy	0.637 (0.000)	0.609 (0.000)	0.665 (0.000)
Spain	0.892 (0.000)	0.793 (0.000)	0.616 (0.000)
France	0.551 (0.046)	0.611 (0.000)	0.467 (0.009)
UK	0.728 (0.001)	0.891 (0.000)	0.856 (0.000)

p-values are shown in parentheses.

Looking at the DCC and sliding window correlation results with raw and MA-shifted data for fever, cough, and dyspnea symptoms, we found that: First, the DCC model proved a better fit than sliding windows correlation models during the first wave of the pandemic. Second, high fit periods for DCC coefficients (r ≳0.90) were different in each country.

**Figure 3:**
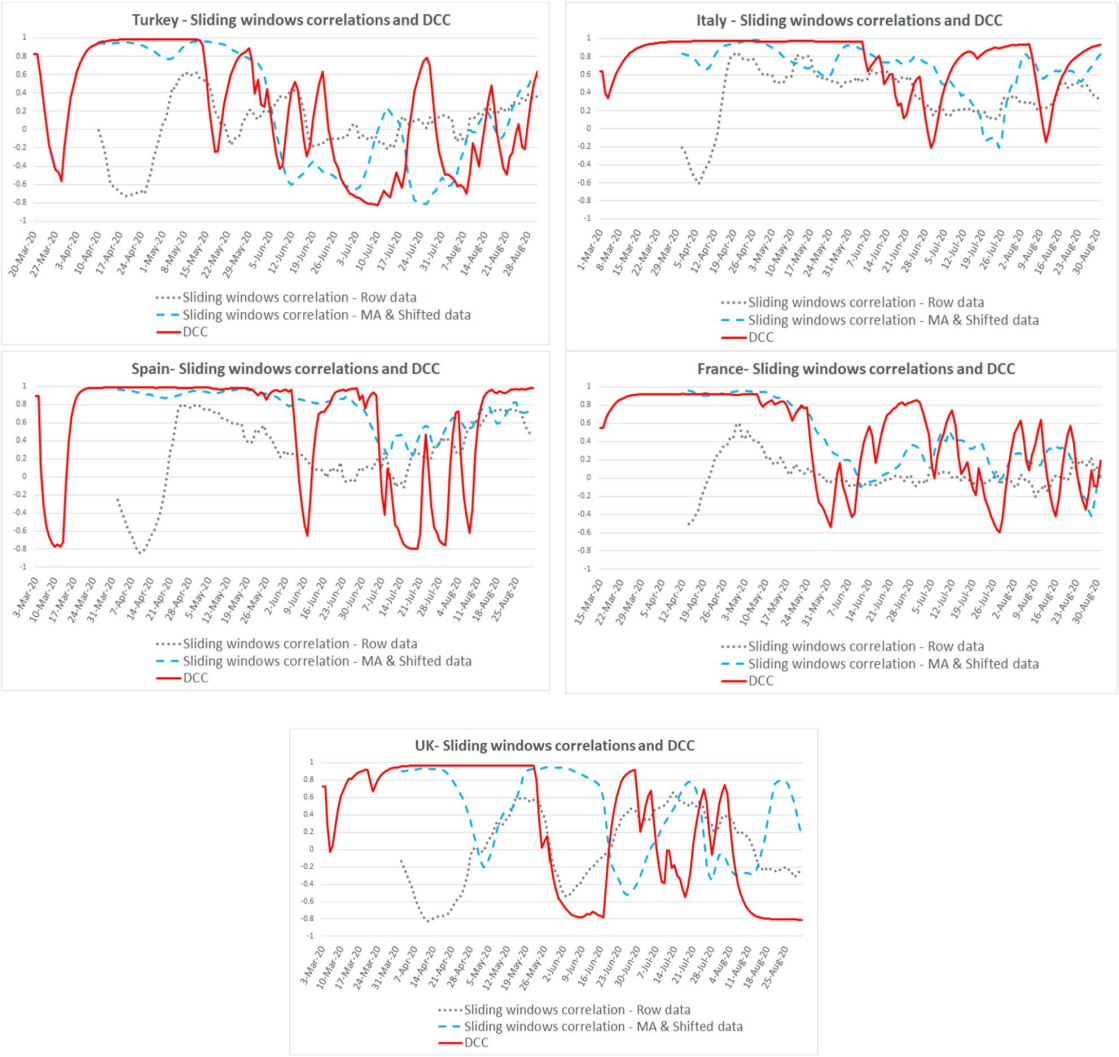
Correlations between fever symptom search and new cases.

For fever, the high fit period is April 10–May 14 for Turkey, March 31–June 5 for Italy, April 2–June 4 for Spain, April 14–May 7 for France, April 18–June 21 for the UK (see Fig. 3 for details).

**Figure 4:**
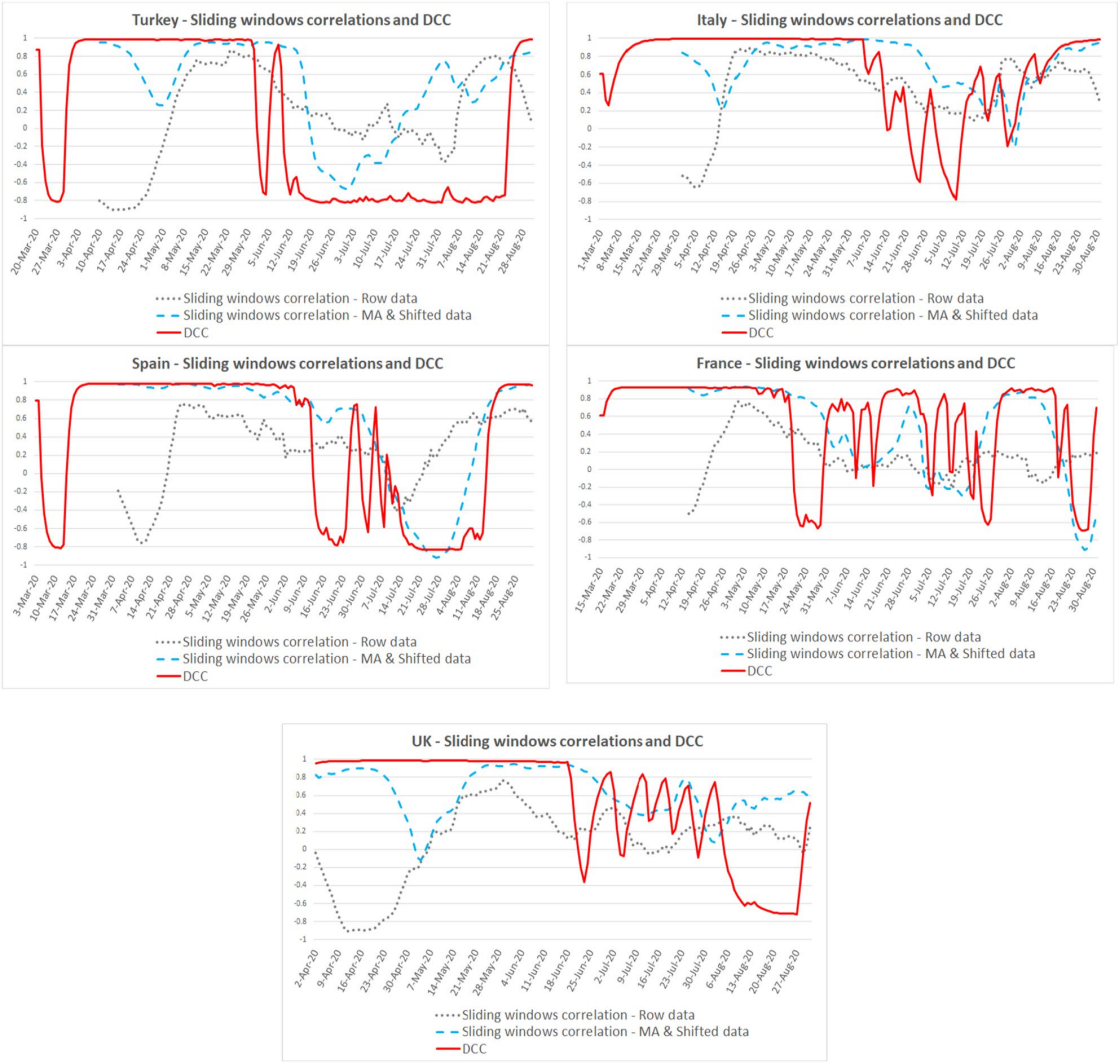
Correlations between cough symptom search and new cases.

For cough, the high fit period (r ≳0.90) is April 10–May 14 for Turkey, March 31–June 5 for Italy, April 2–June 5 for Spain, April 14–May 7 for France, April 18–June 18 for the UK, cough symptom search fit is the highest in the UK (see Fig. 4 for details).

**Figure 5:**
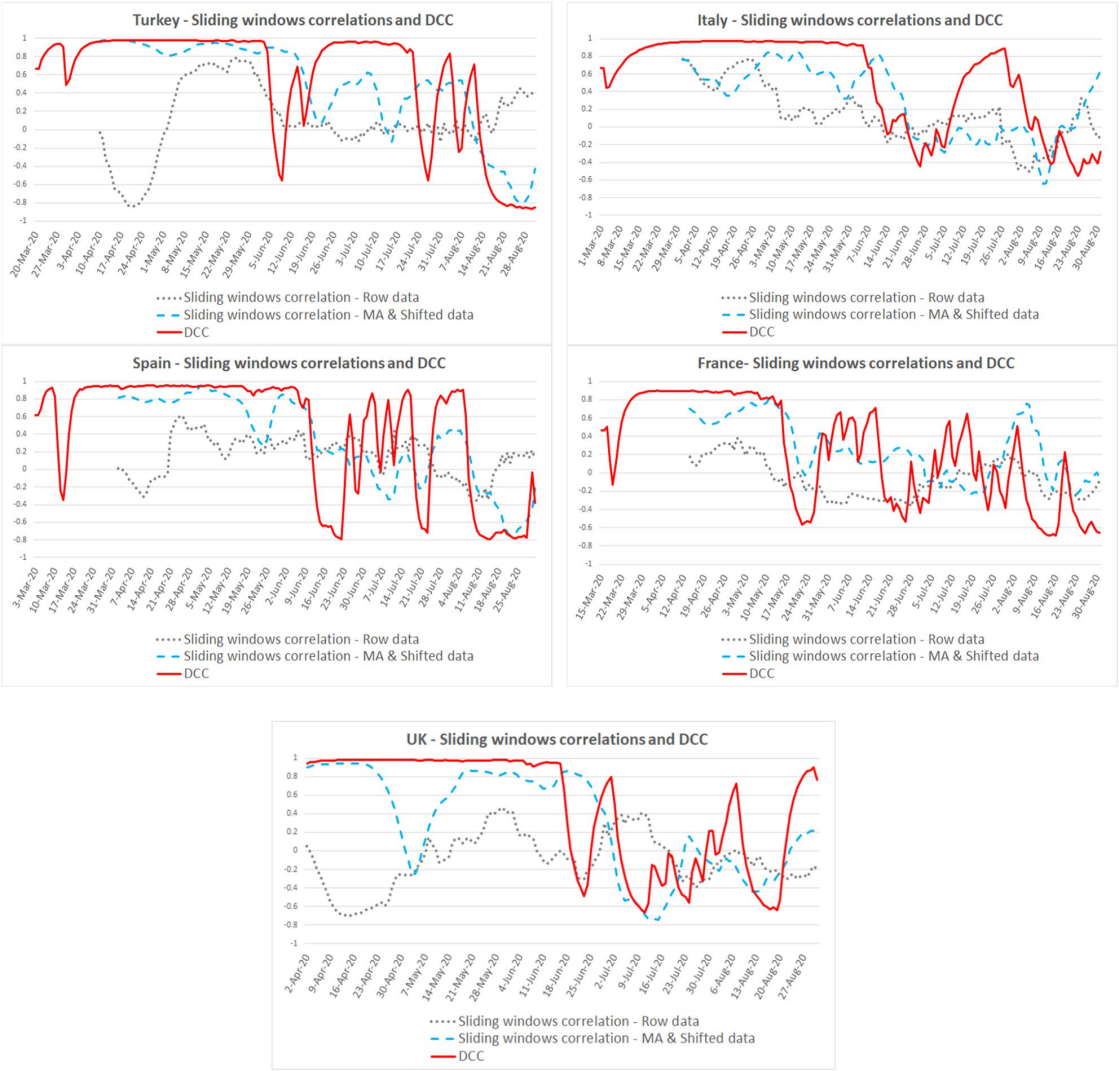
Correlations between dyspnea symptom search and new cases.

For dyspnea, the DCC coefficient fluctuates after the pandemic's first wave. The high fit period (r ≳0.90) is April 10–June 4 for Turkey, March 31–June 5 for Italy, April 2–June 5 for Spain, April 14–May 7 for France, and April 18–June 16 for the UK (see Fig. 5 for details).

## Discussion

This study shows that for three pulmonary symptoms (fever, cough, and dyspnea), google search interest is correlated with COVID-19 new cases utilizing a DCC model. We also demonstrated that the DCC model's performance is better than the sliding windows correlation for data from the first wave of COVID-19 pandemics. Our findings suggest that monitoring Google interest using GT would provide valuable information to produce preventive and intervention related programming.

Previously, Asseo et al. examined the relationship between smell and taste loss symptoms of COVID-19 Google searches and new COVID-19 cases. They employed a sliding window correlation for time frames of one month between March 4-August 25, 2020. The authors could not find stable correlations between taste and smell loss searches and new cases for Italy and the US during the pandemics' first wave. However, they observed that this link fluctuates over time and concluded that the correlation between searches of novel symptoms of infectious disease and the number of new cases fluctuates and decreases over time^25^. We found similar fluctuations. However, we observed much less fluctuation in our data set during the pandemic's first wave. Fluctuations did increase, and correlations decreased as the western hemisphere moved into the summer period.

Lippi et al. investigated the relationship between the volume of Google searches for the most frequent symptoms (fever, cough, and dyspnea) of SARS-CoV-2 infection and new cases using the Spearman correlation test. They did not find a significant correlation between cough/fever and new cases, respectively, but did detect a significant correlation for dyspnea. However, the correlation between newly diagnosed COVID-19 cases and “cough” and “fever” search terms became statistically significant with a 3-week delay. This study used a standard three-week time period and failed to see correlations in several symptoms^15^. However, we used symptom and country-specific time periods ranging from 8–24 days. This dynamic approach helped reveal the correlation in a more fine-tuned manner.

Most studies used conventional correlation methods, and they could not observe the time-varying co-movement between GT symptom search and new case emergence^17,20^. It is important to detect correlations in different periods, such as wave periods in pandemics. The time-varying correlations approach allows to monitor this co-movement in different periods and provides us with multiple correlation indexes. The DCC model is such a model that performs better than other time-varying correlation approaches such as the sliding windows correlation^35^. Thus, we used DCC to find time-varying correlations between pulmonary symptoms and new COVID-19 cases.

Previous studies selected a fixed day for each of the countries^14,17,19^, but the research presented here shows that a heuristic approach (RMSE) results in different shift days for each country's GT symptom. Our results illustrate that Italy, France, and Spain have a shorter time delay ranging from 8 to 21 days; UK and Turkey have a range of 18 to 24 days from symptom search to new case report. These differences may be due to variations in peoples' web search reaction to pulmonary symptoms or the procurement of PCR test results and processing of the test results in each country. Attention paid to higher risk symptoms such as dyspnea may indicate the need for hospitalization, which is of distinct importance.

DCC analysis methods show that fever, cough, and dyspnea symptoms correlate well with new cases during the first wave of the pandemic. However, by May 2020, many fluctuations in correlations begin to appear. We suggest that these fluctuations can result for various reasons independent of the modelling used: symptoms may become too well known, testing of non-symptomatic cases may have become more common, the definition of new case reporting may have changed. New symptoms of interest related to COVID-19 may also have emerged, such as loss of taste, backache, etc. Constant monitoring of the public interest in GT is very important to formulate relevant search models. Yet, we suggest that the modeling approach will remain relevant.

One limitation of this study is the terms selected to carry out the searches. Local/colloquial use of terms was not included, which may have affected the results by limiting the scope. The second limitation is in the selection of the data sources. Though "Google" is the most used web-based search engine, the use of different search tools like "Yahoo," "Msn," or "Yandex" may have led to more accurate assessments of public interest. New case reports were based on the WHO database, and the case reporting protocols may have changed during the pandemic. The third limitation is about lack of causal inference; it would be unwise to interpret our findings to indicate a direct causal relationship between search interest and COVID-19 cases. Mass media coverage of the pandemic may have shifted the GT results towards an increase in COVID-related searches. This increased general interest in the media about COVID-19 during the studied time period may have created some level of spurious correlation^10^.

The 2020 COVID-19 pandemic is the largest global public health challenge of this century. Our findings reveal that pulmonary symptom queries are crucial early signs for emerging epidemics. For example, dyspnea search interest may signal potential hospitalization and the need for intensive care. Policymakers are advised to pay attention to and utilize these search interests to plan preventive and/ or intervention strategies. Monitoring search terms may also help understand the populace's lay beliefs and worries, revealing the need for further guidance. Our results may be of particular importance as we approach the vaccination period with an already existing anti-vaccination movement in place.

## Data Availability

The COVID-19 datasets are obtained from WHO Coronavirus Disease (COVID-19) Dashboard COVID (https://covid19.who.int/), and query dataset analyzed on the “Google Trends” page (https://trends.google.com/trends/), respectively.
